# Violence against women, Espírito Santo, Brazil

**DOI:** 10.1590/S1518-8787.2017051006815

**Published:** 2017-03-29

**Authors:** Franciele Marabotti Costa Leite, Maria Helena Costa Amorim, Fernando C Wehrmeister, Denise Petrucci Gigante

**Affiliations:** I Programa de Pós-Graduação em Epidemiologia. Universidade Federal de Pelotas. Pelotas, RS, Brasil; II Departamento de Enfermagem. Programa de Pós-Graduação em Enfermagem. Universidade Federal do Espírito Santo. Espírito Santo, ES, Brasil; III Departamento de Medicina Social. Programa de Pós-Graduação em Epidemiologia. Universidade Federal de Pelotas. Pelotas, RS, Brasil; IV Departamento de Nutrição. Programa de Pós-Graduação em Epidemiologia. Universidade Federal de Pelotas. Pelotas, RS, Brasil

**Keywords:** Battered Women, Violence Against Women, Spouse Abuse, Intimate Partner Violence, Domestic Violence, Family Relations, Socioeconomic Factors, Cross-Sectional Studies

## Abstract

**OBJECTIVE:**

To estimate the prevalence and factors associated with psychological, physical and sexual violence in women victims of intimate partner violence assisted in the primary care services.

**METHODS:**

This is a cross-sectional study, conducted in 26 health units in Vitória, State of Espírito Santo, from March to September 2014. We interviewed 991 women aged 20-59 years. To classify the psychological, physical and sexual violence, the World Health Organization instrument on violence against women was used and a questionnaire to investigate the sociodemographic, behavioral characteristics, and the women’s family and life history was developed. The statistical analyzes used were Poisson regression, Fisher’s exact test and Chi-square.

**RESULTS:**

The prevalence we observed were psychological 25.3% (95%CI 22.6–28.2); physical 9.9% (95%CI 8.1–11.9) and sexual 5.7% (95%CI 4.3–7.3). Psychological violence remained associated with education, marital status, maternal history of intimate partner violence, sexual violence in childhood and drug use, while physical assault was related to age, education, marital status and maternal history of intimate partner violence. Sexual violence occurred the most among women with low income, and victims of sexual violence in childhood.

**CONCLUSIONS:**

Psychological, physical and sexual violence showed highly frequency among women assisted by primary care services. Sociodemographic and behavioral factors, personal experiences, and maternal violence influence the phenomenon.

## RESUMO

**OBJETIVO:**

Estimar a prevalência e os fatores associados às violências psicológica, física e sexual nas mulheres vítimas de violência perpetrada pelo parceiro íntimo atendidas nos serviços de atenção primária.

**MÉTODOS:**

Estudo transversal, realizado em 26 unidades de saúde do município de Vitória, no Espírito Santo, de março a setembro de 2014. Foram entrevistadas 991 usuárias de 20 a 59 anos. Para classificar as violências psicológica, física e sexual foi utilizado o instrumento da Organização Mundial de Saúde sobre violência contra a mulher e um questionário foi elaborado para investigar as características sociodemográficas, comportamentais e de história familiar e de vida da mulher. As análises estatísticas utilizadas foram: regressão de Poisson, teste exato de Fisher e Qui-quadrado.

**RESULTADOS:**

As prevalências observadas foram: psicológica 25,3% (IC95% 22,6–28,2); física 9,9% (IC95% 8,1–11,9) e sexual 5,7% (IC95% 4,3–7,3). A violência psicológica manteve-se associada à escolaridade, situação conjugal, histórico materno de violência por parceiro íntimo, violência sexual na infância e ter feito uso de drogas, enquanto a agressão física esteve relacionada à idade, escolaridade, situação conjugal e a história materna de violência por parceiro íntimo. A violência sexual foi mais frequente nas mulheres de menor renda e que sofreram abuso sexual na infância.

**CONCLUSÕES:**

As violências psicológica, física e sexual apresentaram alta magnitude entre as mulheres usuárias dos serviços de atenção primária de saúde. Fatores sociodemográficos, comportamentais e experiências pessoal e materna de violência influenciam a ocorrência do fenômeno.

## INTRODUCTION

Violence is a complex and multicausal phenomenon. According to the World Health Organization (WHO), it consists of the intentional use of physical force or power, real or threatened, against themselves, another person, a group or a community that results, or is likely to result, in injury, death, psychological damage, developmental disability and deprivation^24^. Globally recognized as a public health problem, violence committed against women usually occurs in the private sphere and the intimate partner is the main perpetrator. Thus, this fact refers women to an intimate relationship with physical assault, sexual coercion, psychological abuse and controlling behaviors^13^.

Violence can lead to significant harm to the victim, even death. In the Map of Violence Brazil ranked fifth among the countries with the highest homicide rate per 100,000 women in 2013. Espírito Santo ranked second among the Brazilian states, and the municipality of Vitória has the highest risk of death of women for homicides^23^.

Violence against women is a complex network of associations and may show variations in different places^6^. This phenomenon involves the interaction of individual, relational, social, cultural and environmental factors^12^. Aspects related to gender influence this abuse, since it is linked to the unequal position of women in relationships, and the male “right” to control over feminine goods and behaviors, so that when women challenge this control or man cannot maintain it, violence happens^9^.

Between 15% and 71% of women have experienced some kind of physical or sexual violence, or both, committed by the intimate partner, as has been reported in a number of countries by a WHO multi-country study on women’s health and domestic violence against women^6^; the lowest proportions were found in Japan and the highest in Ethiopia^7^. Brazil participated in this study by researching São Paulo, SP, and Zona da Mata, PE, with a prevalence of violence of 29% and 37%, respectively^6^.

Most victims of physical marital violence do not seek any kind of help, and when they do, they first seek for those closest to them, followed by institutions such as police, specific services for victims of domestic violence, and health professionals^4^. In this context, health units constitute a privileged space for tracking cases of violence. In this environment, frequent, constant and legitimized access to women throughout their lives occurs. The health unit provides a closer relationship with the community, addressing the common health problems that may often be associated with violence against women^21^.

Thus, this study aimed to estimate the prevalence and factors associated with psychological, physical and sexual violence in women assisted in primary care services victims of violence perpetrated by their intimate partner.

## METHODS

This is a cross-sectional study, carried out in 26 Health Units (HU) in Vitória, State of Espírito Santo. Participants were HU users between 20 and 59 years old, who had an intimate partner in the 12 months prior to the interview date. At the beginning of the interview, the woman was asked whether she had an intimate partner, male, or had in the past 12 months. The intimate partner was defined as the companion or former companion, independent of formal marriage, and current boyfriends, as long as they were having intercourse. Data collection took place from March to September 2014. We approached the eligible women at the health units facilities and explained them the survey. The interview took place in a health unit private place, with only the interviewee and the interviewer. All the women participated in training to interview standardization and instruments application. In addition to the interviewers, the survey counted on the supervisors, who supported the fieldwork, with daily monitoring and the accomplishment of quality control of the interviews. Research questionnaires were used in 10% of the women interviewed in each health unit. The research team prepared a detailed folder containing the main services for women victims of violence, distributed it to the participants as educational, and support material, regardless of reports of violence. An association was established with the nucleus of assistance to victims of violence in Vitória for referral, when necessary.

Considering that Vitória ranked first in female homicide in 2010^23^, the highest prevalences of violence against women identified in the literature (56%)^14^ were estimated for the sample size calculation. Thus, to calculate the sample size we considered a margin of error of five percentage points and a 95%CI acceptable. To study the association with risk factors, we considered 95%CI, 80% power and 1:1 exposed/unexposed ratio. We added 10% for possible losses and 30% for adjusted analyzes. Thus, we should include 998 women selected in each of the 26 health units of Vitória, by means of a sample proportional to the number of women, aged 20 to 59 years, registered in each unit. The study had 991 women participating (99.3% of the sample). Seven women declined to participate.

The three types of violence against women (psychological, sexual or physical) were the dependent variables in this study. They all occurred when the woman answered yes to one of the items on the instrument. To identify the outcomes, the reduced version of the original WHO questionnaire on violence against women was used. This questionnaire is validated to Brazil and aims to discriminate the different forms of violence against women in their psychological, physical and sexual domains. This instrument has 13 questions related to violence, can discriminate the different forms in diverse social contexts, and is comprehensive and relatively short. This instrument has high internal consistency, presented by the *Cronbach coefficients (*mean of 0.88)^18^.

An instrument was developed containing the sociodemographic characteristics to obtain the independent variables age (in complete years and categorized by decades); self-reported skin color (according to the Brazilian Institute of Geography and Statistics [IBGE]): white, black and mixed. We excluded women who declared themselves as indigenous or yellow for being an underrepresented group and no inference of the results was possible; schooling (in complete years of study: up to eight, nine, or more years); marital status (married, single, divorced or separated; and in consensual union, that is, living with the partner, but not legally married); religion (Catholic or Protestant – yes; no) and family income (the sum of the monthly income in Reais of each of the residents, which was later distributed in tertiles). Regarding family and life experience, the woman was asked the following questions, respectively: “Has your mother ever suffered any violence from the intimate partner?” and “Have you ever suffered sexual violence in childhood?” each variable presented in a dichotomous way (yes; no). The behavioral variables were doses of alcoholic drink (none; up to two; or more than two). On average, one dose was a 350 ml can of beer, a 90 ml of wine glass, a 30 ml distillate dose, a can or a small bottle of any *ice* beverage; two categories of smoking: smokers – smoked at least one cigarette per day – non-smokers, including ex-smokers); and history of drug use (drug use ever in life – yes; no).

Pearson’s Chi-square or Fisher’s exact tests for contingency tables with a small number of observations were used in the descriptive analyzes to frequencies and their confidence intervals. According to the hierarchical model (Figure), the adjusted analysis was performed, controlling for possible confounding factors. For inclusion in the multiple model, a p value was not limited to avoid the exclusion of potentially confounding variables, and the variables that had statistical significance (p < 0.05) were maintained in the model. We conducted such analyzes using Poisson regression, and prevalence ratio as an effect measure. The analyzes were performed with the Stata 13.0 statistical package.

**Figure:**
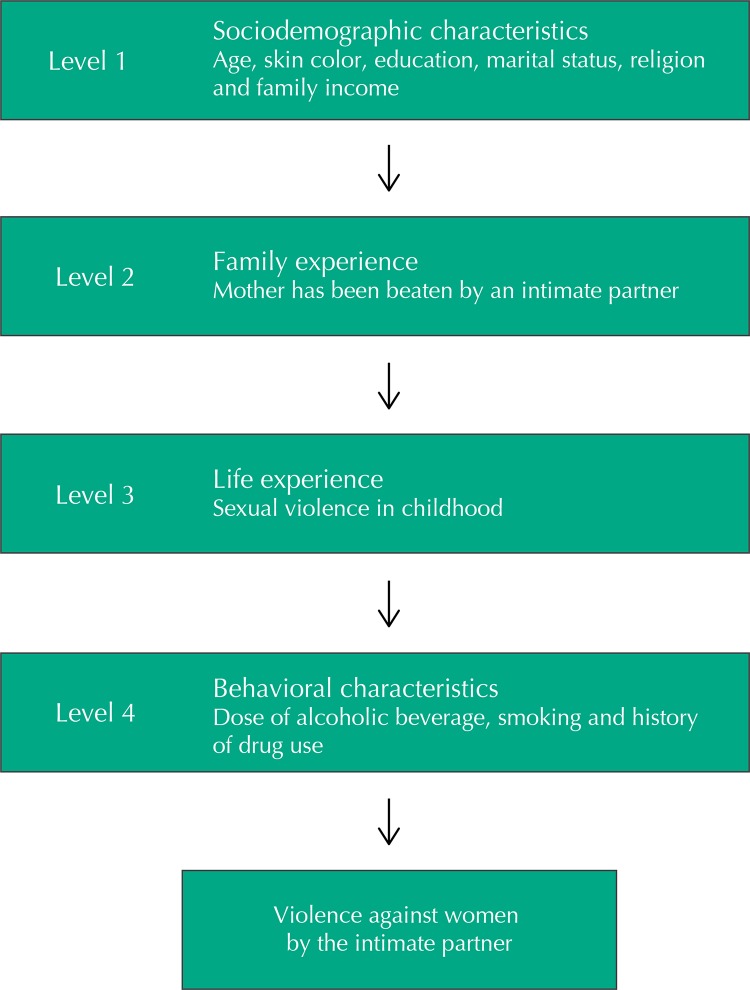
Hierarchical model of the relationship between risk factors for the outcome of violence against women by the intimate partner.

The Research Ethics Committee of the Federal University of Espírito Santo approved the study (Opinion 470.744/2013). Participants signed free and informed consent form.

## RESULTS

Psychological violence was the most frequent, with a prevalence of 25.3% (95%CI 22.6–28.2), followed by physical violence (9.9%; 95%CI 8.1–11.9). Sexual violence had the lowest prevalence (5.7%; 95%CI 4.3–7.3).

At the time of the interview most women were under 40 years, declared to be mixed, and had nine or more years of education. Almost 35% had family income up to R$1,500.00. About half of them said to be Catholic or Protestant, and 30% reported that their mother had been beaten up by an intimate partner, while 10% had experienced sexual violence in childhood. Most reported consuming fewer than two doses of alcohol and denied being a smoker or having a history of drug use. On the other hand, about one in 10 women reported consuming five or more doses of alcohol, being a smoker and having a history of drug use (Table 1).

**Table 1:** Prevalence of violence against woman by the intimate partner in the last 12 months, according to sociodemographic and behavioral characteristics, family and life experience. Vitória, State of Espírito Santo, March to September 2014.

Sociodemographic characteristics	n	%	Psychological violence			Physical violence			Sexual violence		
		
			%	95%CI	p	%	95%CI	p	%	95%CI	p
Age (years)					0.321			0.137			0.313
20–29	285	28.8	24.6	19.9–29.9		12.3	8.9–16.6		3.9	2.1–6.8	
30–39	306	30.9	28.8	23.9–34.1		11.1	8.0–15.2		5.9	3.7–9.1	
40–49	225	22.7	21.8	16.8–27.7		7.6	4.7–11.8		5.8	3.4–9.7	
50–59	175	17.6	25.1	19.2–32.1		6.9	3.9–11.7		8.0	4.8–13.1	
Skin color^a^					0.598			0.051			0.933
White	215	22.5	23.7	18.5–29.9		5.6	3.2–9.6		6.0	3.5–10.1	
Mixed	503	52.5	26.6	22.9–30.7		10.9	8.5–14.0		5.4	3.7–7.7	
Black	239	25.0	23.8	18.8–29.7		11.7	8.2–16.5		5.4	3.2–9.1	
Education (years of education)					0.000			0.011			0.019
0–8	303	30.6	33.0	27.9–38.5		13.5	10.1–17.9		8.2	5.6–11.9	
≥ 9	688	69.4	21.9	19.0–25.2		8.3	6.4–10.6		4.5	3.1–6.3	
Family income (tertiles)					0.025			0.004			0.007
First	343	34.6	30.3	25.7–35.4		13.7	10.4–17.8		8.7	6.2–12.2	
Second	318	32.1	23.9	19.5–28.9		9.7	6.9–13.5		4.7	2.8–7.7	
Third	330	33.3	21.5	17.4–26.3		6.1	3.9–9.2		3.3	1.8–5.9	
Marital status					0.005			0.005			0.144^b^
Married	438	44.2	24.2	20.4–28.4		8.0	5.8–10.9		4.8	3.1–7.2	
Consensual union	295	29.8	29.5	24.5–35.0		12.2	8.9–16.5		7.8	5.2–11.5	
Single	238	24.0	20.2	15.5–25.8		8.8	5.8–13.2		4.2	2.3–7.6	
Divorced/Separated	20	2.0	50.0	28.9–71.1		30.0	13.8–53.4		10.0	2.4–33.2	
Catholic					0.177			0.757			0.713
Yes	419	42.3	23.1	19.3–27.4		9.5	7.1–12.8		6.0	4.0–8.7	
No	572	57.7	26.9	23.4–30.7		10.1	7.9–12.9		5.4	3.8–7.6	
Protestant					0.024			0.042			0.286
Yes	480	48.4	28.5	24.7–32.7		11.9	9.3–15.1		6.5	4.6–9.0	
No	511	51.6	22.3	18.9–26.1		8.0	5.9–10.7		4.9	3.3–7.1	

Family and life experiences											

Mother has been beaten by a partner^c^					0.000			0.001			0.501
Yes	313	31.6	34.5	29.4–399		14.1	10.6–18.4		6.4	4.1–9.7	
No	585	59.0	19.1	16.1–22.5		7.0	5.2–9.4		5.3	3.7–7.4	
Sexual violence in childhood					0.000			0.102			0.003
Yes	121	12.2	42.1	33.6–51.1		14.0	8.9–21.5		11.6	6.9–18.6	
No	870	87.8	23.0	20.3–25.9		9.3	7.5–11.4		4,8	3.6–6.5	

Behavioral characteristics											

Dose of alcoholic beverage (ml)					0.584			0.030			0.271
< 2,0	734	74.1	24.6	21.7–27.8		8.8	7.0–11.0		5.5	4.1–7.3	
2.0–4.9	136	13.7	26.4	18.2–36.7		10.3	5.4–18.8		9.2	4.6–17.4	
≥ 5,0	121	12.2	28.9	21.5–37.7		16.5	10.9–24.3		4.1	1.7–9.6	
Smoke					0.015			0.000			0.212
Yes	109	11.0	34.9	26.5–44.3		20.2	13.6–28.8		8.3	4.3–15.2	
No	882	89.0	24.1	21.4–27.1		8.6	6.9–10.7		5.3	4.0–7.0	
History of drug use					0.002			0.000			0.371
Yes	106	10.7	37.7	29.0–47.4		23.6	16.4–32.6		7.5	3.8–14.4	
No	885	89.3	23.8	21.1–26.8		8.2	6.6–10.2		5.4	4.1–7.1	

^a^ n = 957

^b^ Fisher’s exact test.

^c^ n = 898

The highest frequency of psychological violence was among women with up to eight years of education, belonging to the lower income group, separated or divorced, and Protestant (Table 1). Higher prevalence of psychological violence occurred in those with family violence or in childhood and with a history of drug use. The highest prevalence of physical violence occurred in self-reported black women, with lower schooling and family income, separated or divorced, and Protestant. It was also more frequent in women with a maternal history of intimate partner violence, drug use, and alcohol consumption. Higher prevalence of sexual violence occurred in those with lower education and income and who suffered sexual violence in childhood (Table 1).

Psychological violence was associated with education, marital status, maternal history of intimate partner violence, sexual violence in childhood, and drug use in the adjusted analysis. On average, psychological violence was 45% more frequent among those with lower education, when compared to those with nine years or more of education and about twice as many in divorced or separated as compared to married women. The prevalence of psychological violence was 70% higher in women whose mother suffered some type of intimate partner violence or who were sexually abused in childhood. History of drug use was associated with psychological violence, with a prevalence ratio of 1.35 (Table 2).

**Table 2:** Gross and adjusted analysis of the effects of sociodemographic, behavioral variables and family and life experience on intimate partner psychological violence in the last 12 months. Vitória, State of Espírito Santo, March to September 2014.

Sociodemographic characteristics	n	Crude analysis			Adjusted analysis		
	
		Crude PR	95%CI	p	Adjusted PR	95%CI	p
Age (years)				0.323			0.183
20–29	285	1			1		
30–39	306	1.17	0.89–1.53		1.11	0.85–1.45	
40–49	225	0.89	0.64–1.22		0.79	0.57–1.10	
50–59	175	1.02	0.74–1.42		0.95	0.68–1.34	
Color^a^				0.600			0.805
White	215	1			1		
Mixed	503	1.12	0.85–1.49		0.95	0.71–1.27	
Black	239	1.00	0.72–1.40		0.89	0.64–1.25	
Education (years of education)				0.000			0.001
0–8	303	1.50	1.21–1.86		1.45	1.17–1.80	
9–11	688	1			1		
Family income (tertiles)				0.024			0.361
First	343	1.41	1.08–1.83		1.14	0.86–1.53	
Second	318	1.11	0.83–1.48		0.96	0.71–1.29	
Third	330	1.			1		
Marital status				0.002			0.006
Married	438	1			1		
Consensual union	295	1.22	0.96–1.55		1.17	0.92–1.49	
Single	238	0.83	0.61–1.13		0.85	0.63–1.14	
Divorced/Separated	20	2.07	1.29–3.30		1.97	1.24–3.13	
Catholic				0.180			0.727
Yes	419	0.86	0.69–1.07		0.95	0.73–1.24	
No	572	1			1		
Protestant				0.025			0.079
Yes	480	1.28	1.03–1.59		1.22	0.98–1.52	
No	511	1			1		

Family and life experiences							

Mother has been beaten by a partner^c^				0.000			0.000
Yes	313	1.80	1.44–2.26		1.71	1.35–2.15	
No	585	1			1		
Sexual violence in childhood				0.000			0.000
Yes	121	1.83	1.44–2.33		1.74	1.32–2.28	
No	870	1			1		

Behavioral characteristics							

Dose of alcoholic beverage (ml)				0.574			0.837
< 2.0	734	1			1		
2.0–4.9	136	1.07	0.74–1.55		1.13	0.76–1.67	
≥ 5.0	121	1.17	0.86–1.59		1.02	0.72–1.44	
Smoke				0.011			0.203
Yes	109	1.44	1.09–1.91		1.23	0.89–1.69	
No	882	1			1		
History of drug use				0.001			0.049
Yes	106	1.58	1.21–2.08		1.35	1.01–1.83	
No	885	1			1		

^a^ n = 957

^b^ n = 898

For physical violence (Table 3), age, education and marital status significantly associated with physical violence in the adjusted model. Older women (40 to 59 years of age) suffered less physical violence compared to those aged 20 to 29 years, while a higher prevalence of physical violence victimization occurred on average in women with lower education who declared to be divorced or separated or whose mother was the victim of violence. Women with a history of drug use had a 2.4 times greater prevalence of physical violence by the intimate partner (Table 3).

**Table 3:** Crude and adjusted analysis of the effects of sociodemographic, behavioral, family and life experience on physical violence by the intimate partner in the last 12 months. Vitória, State of Espírito Santo, March to September 2014.

Sociodemographic characteristics	n	Crude analysis			Adjusted analysis		
	
		Crude PR	95%CI	p	Adjusted PR	95%CI	p
Age (years)				0.149			0.046
20–29	285	1			1		
30–39	306	0.90	0.58–1.41		0.86	0.55–1.35	
40–49	225	0.61	0.35–1.07		0.52	0.29–0.92	
50–59	175	0.56	0.30–1.05		0.50	0.26–0.94	
Color^a^				0.063			0.168
White	215	1			1		
Mixed	503	1.96	1.07–3.58		1.58	0.86–2.93	
Black	239	2.10	1.09–4.02		1.86	0.98–3.54	
Education (years of education)				0.011			0.003
0–8	303	1.63	1.12–2.38		1.79	1.21–2.66	
≥ 9	688	1			1		
Family income (tertiles)				0.005			0.418
First	343	2.26	1.37–3.73		1.42	0.82–2.46	
Second	318	1.60	0.94–2.76		1.18	0.67–2.09	
Third	330	1			1		
Marital status				0.003			0.009
Married	438	1			1		
Consensual union	295	1.53	0.98–2.37		1.27	0.81–1.99	
Single	238	1.10	0.66–1.85		1.04	0.63–1.74	
Divorced/Separated	20	3.75	1.79–7.88		3.49	1.65–7.35	
Catholic				0.758			0.232
Yes	419	0.94	0.64–1.38		1.30	0.84–2.01	
No	572	1			1.		
Protestant				0.044			0.116
Yes	480	1.48	1.01–2.17		1.37	0.92–2.03	
No	511	1			1		

Family and life experiences							

Mother has been beaten by a partner^c^				0.001			0.021
Yes	313	2.00	1.34–3.00		1.63	1.08–2.46	
No	678	1			1		
Sexual violence in childhood				0.098			0.203
Yes	121	1.51	0.93–2.46		1.40	0.83–2.37	
No	870	1			1		

Behavioral characteristics							

Dose of alcoholic beverage (ml)				0.027			0.738
< 2.0	734	1			1		
2.0–4.9	136	1.17	0.61–2.27		1.25	0.62–2.51	
≥ 5.0	121	1.87	1.18–2.97		1.16	0.67–2.01	
Smoke				0.001			0.076
Yes	109	2.34	1.52–3.60		1.57	0.95–2.60	
No	882	1			1		
History of drug use				0.000			0.000
Yes	106	2.85	1.90–4.30		2.40	1.54–3.76	
No	885	1			1		

^a^ n = 957

^b^ n = 898

Only the family income remained associated with sexual violence by the intimate partner, disregarding the education gross effect (Table 4). Women in the lowest tertile of family income (up to R$1,500.00/month) were victims of sexual violence by the partner about three times more than the women of the largest tertiles. Although the woman’s age was not significantly associated with sexual violence, the 50–59 age group had an average prevalence 2.5 times higher than the 20–29 age group. Sexual violence was about twice as prevalent in the group that experienced sexual violence in childhood.

**Table 4:** Crude and adjusted analysis of the effects of sociodemographic, behavioral, family and life experience on sexual violence by the intimate partner in the last 12 months. Vitória, State of Espírito Santo, March to September 2014.

Sociodemographic characteristics	n	Crude analysis			Adjusted analysis		
	
		Crude PR	95%CI	p	Ajusted PR	95%CI	p
Age (years)				0.325			0.113
20–29	285	1			1		
30–39	306	1.52	0.73–3.17		1.56	0.75–3.22	
40–49	225	1.50	0.68–3.28		1.69	0.78–3.68	
50–59	175	2.07	0.96–4.46		2.53	1.20–5.37	
Color^a^				0.933			0.484
White	215	1			1		
Mixed	503	0.89	0.47–1.69		0.68	0.35–1.30	
Black	239	0.90	0.43–1.90		0.70	0.34–1.48	
Education (years of education)				0.020			0.487
0–8	303	1.83	1.10–3.05		1.21	0.70–2.08	
≥ 9	688	1			1		
Family income (tertiles)				0.009			0.003
First	343	2.62	1.34–5.15		2.99	1.52–5.90	
Second	318	1.41	0.66–3.03		1.59	0.74–3.40	
Third	330	1			1		
Marital status				0.195			0.239
Married	438	1			1		
Consensual union	295	1.63	0.92–2.88		1.56	0.87–2.80	
Sinlge	238	0.88	0.42–1.83		0.80	0.38–1.68	
Divorced/Separated	20	2.08	0.52–8.29		1.72	0.48–6.22	
Catholic				0.713			0.875
Yes	419	1.10	0.66–1.84		1.05	0.54–2.05	
No	572	1			1		
Protestant							
Yes	480	1.32	0.79–2.20	0.288	1.23	0.74–2.04	0.427
No	511	1			1		

Family and life experiences							

Mother has been beaten by a partner^c^				0.501			0.763
Yes	313	1.20	0.70–2.08		1.09	0.63–1.87	
No	585	1			1		
Sexual violence in childhood				0.003			0.002
Yes	121	2.40	1.35–4.26		2.43	1.38–4.29	
No	870	1			1		

Behavioral characteristics							

Dose of alcoholic beverage (ml)				0.272			0.111
< 2.0	734	1			1		
2.0–4.9	136	1.67	0.81–3.44		1.94	0.94–4.00	
≥ 5.0	121	0.75	0.30–1.86		0.68	0.27–1.68	
Smoke				0.210			0.328
Yes	109	1.55	0.78–3.07		1.41	0.70–2.84	
No	882	1			1.00		
History of drug use				0.369			0.857
Yes	106	1.39	0.68–2.86		1.07	0.51–2.25	
No	885	1			1		

^a^ n = 957

^b^ n = 898

## DISCUSSION

The results show a higher prevalence of psychological violence among women users of primary care services, followed by physical and sexual violence. Psychological and physical violence were associated, even after adjustment, with education, marital status, maternal history of intimate partner violence and drug use. Sexual violence only remained associated with family income.

Violence against women takes place throughout history in virtually every country with the most diverse economic and political regimes. The magnitude of violence is different and is more common in countries with a predominantly male culture, and less frequent in cultures seeking egalitarian solutions to gender differences^3^.

Brazil’s economic landscape changed profoundly from the mid-19th century until after the First World War. This brought the contact with behaviors and values of other countries, which started being confronted with the patriarchal customs still valid, although weakened. However, even after 30 years of struggle for equal rights between men and women, there have been countless and cruel episodes of violence against women. The culture of woman subordination to the man from whom she is considered an inalienable and eternal property is suggested^3^.

Psychological violence in Vitória showed higher prevalence when compared to other types of violence, in line with the literature^14^ ^,^ ^17^. Psychological abuse is also often the most neglected and rarely recognized violence. However, its importance should be noted. The aggressor, in his first manifestations, does not use physical violence, but restricts the victim’s freedom, advancing to the embarrassment and humiliation^20^.

Physical violence involves acts of bodily aggression, which is the most obvious^20^. The prevalence this study found was similar to that found in São Paulo, also observing sexual violence^14^. The latter is marked by invisibility for its little recognition as violence. A study carried out with users of the emergency service in Salvador in 2001 showed that women who suffer rape by their partner did not consider the act as sufficient reason to punish the aggressor, especially if it occurred without physical violence^19^.

Violence against women is motivated by inequality expressions based on the sex condition, which begins in the family environment, in which gender relations are established in the model of hierarchical relationships. In some situations those who dominate and who are dominated can receive marks of race, age, class, among others, changing their position in relation to the position of the family nucleus^1^.

When analyzing the occurrence of violence in relation to the women’s age, the lowest prevalences of physical violence occur among women in the 50–59 age group, when compared to younger women. Older aggressions would be less valued in the report, so that the frequencies of violence in older women would be underestimated, even if violence starts early in most of the time^6^.

Sexual violence would be more prevalent among women aged 50–59 years than among the younger women. Some situations of intimate partner sexual violence are common in our society and, therefore, acceptable and not recognized as violence^19^. Women often give in to the partner’s desire to no contradict him or at times by understanding that it is an obligation in the relationship between husband and wife^19^.

We observed violence against women with lower education and income. These variables are important in the study of intimate partner violence. Lower social support represents a greater risk, because women tend to submit more often to the perpetrator because of the lack of opportunity to fight and face violence^19^. However, there was no association of any type of violence with skin color or religion, as in other studies^19^ ^,^ ^22^.

Divorced or separated women had higher prevalences of suffering psychological and physical violence by their partner in the last 12 months than married and unmarried women. This finding resembles other studies^6^ ^,^ ^22^, and suggests that these women had previously experienced violent relationships and were able to break free from this situation, breaking the violence cycle^6^.

Although the literature points out that victims of violence are more likely to smoke^5^ and higher consumption of alcohol and drugs^16^, in this study only the history of drug use was associated with psychological and physical violence, as presented in another study^22^.

The association between the report that the mother has suffered some type of violence with psychological and physical violence leads to the reflection on the transgenerational violence transmission, in which it is perpetuated from generation to generation as a normal situation within the family^2^. The involvement in family violence contexts, as a victim or as a witness, enables the establishment of a violent conjugal relationship in adulthood^8^. Moreover, having been a victim of sexual violence in childhood was related to sexual violence by the partner, suggesting that children from contexts of violence may be more likely to show, in their future relationships, the tendency of repetition of lived patterns^15^.

Violence situations lead to serious damage to the victims’ health with regard to physical, sexual, reproductive, emotional, mental and social development well-being of the individual and the family. Immediate and long-term health outcomes associated with these types of violence include physical trauma, unwanted pregnancy, abortion, gynecological complications, sexually transmitted infections, posttraumatic stress disorder, among others^13^. In this context, the research was important considering the violence magnitude among the users. It is a device to foster new practices in health units, such as the discussion of violence and the creation of a space for listening, welcoming and assisting women, to better understand and confront this phenomenon^10^.

Not only women, but also health professionals have difficulty speaking and dealing with violence. Women tend to be silent about violence and generally do not make spontaneous complaints during consultations in the basic health network^14^, unless they receive welcoming and listening conditions^10^. On the other hand, health professionals do not feel empowered to deal with such situations^14^. The team should receive instrumentalization by elaboration and implementation of protocols that aim at an full, interdisciplinary and quality care to the victimized woman^11^.

Possible limitations should be considered, so due to information bias, prevalence may have been underestimated. However, the face-to-face interview made by women, in a private setting, in the health unit may have minimized this bias. As the results refer to the users of the health units, it is possible that women who have suffered violence have been inhibited to attend the health service. However, the results show high prevalence for the three types of violence. If they have been underestimated, greater forces of association could be found with some exposures and outcomes. Also, reverse causality may be present in associations with behavioral and socioeconomic variables. However, the strong associations with behavioral variables point to the need to propose measures that influence exposures and outcomes.

It is also important to identify women who have already suffered some type of violence so that specific measures can be proposed to prevent and deal with intimate partner violence. Despite the cross-sectional design, we detected the effect of previous and familiar experiences of violence, considering that they preceded the violence practiced by the partner.

Data show high victim prevalence, and that sociodemographic, behavioral or life and personal factors may make women more vulnerable to intimate partner violence. We hope that the results contribute to the broadening of the debates and the formulation of strategies for the promotion, prevention, detection and monitoring of violence.
